# Determinants of Implementation of Antimicrobial Stewardship Interventions for Managing Community Adult Acute Respiratory Infections: Qualitative Analysis from the OPTIMAS-GP Study Co-Design Phase

**DOI:** 10.3390/antibiotics14090914

**Published:** 2025-09-11

**Authors:** Margaret Jordan, Mary Burns, Colin Cortie, Janette Radford, Christine Metusela, Judy Mullan, Simon Eckermann, Fiona Williams, Caitlin Keighley, Danielle Mazza, Indra Gajanayake, Stephen Barnett, Andrew Bonney

**Affiliations:** 1 Graduate School of Medicine, Faculty of Science, Medicine and Health, University of Wollongong, Wollongong, NSW 2522, Australia; marybu@uow.edu.au (M.B.); colinc@uow.edu.au (C.C.); metusela@uow.edu.au (C.M.); jmullan@uow.edu.au (J.M.); fionaw@uow.edu.au (F.W.); caitlink@uow.edu.au (C.K.); indra.gajanayake@gmail.com (I.G.); sbarnett@uow.edu.au (S.B.); abonney@uow.edu.au (A.B.); 2 Launceston Clinical School, Tasmanian School of Medicine, University of Tasmania, Launceston, TAS 7250, Australia; j.radford@utas.edu.au; 3 School of Public Health, Faculty of Social Sciences, University of Wollongong, Wollongong, NSW 2522, Australia; seckerma@uow.edu.au; 4 Infectious Diseases, Illawarra Shoalhaven Local Health District, Wollongong Hospital, Wollongong, NSW 2500, Australia; 5 Southern.IML Pathology, 3 Bridge Street, Coniston, Wollongong, NSW 2500, Australia; 6 School of Public Health, Faculty of Medicine, Nursing and Health Sciences, Monash University, Clayton, VIC 3800, Australia; danielle.mazza@monash.edu; 7 Health Care Consumers Association, Chifley, ACT 2606, Australia

**Keywords:** acute respiratory tract infection, antimicrobial resistance, antimicrobial stewardship, C-reactive protein, delayed prescribing, diagnostic stewardship, general practice, point-of-care testing, primary care, shared decision-making

## Abstract

Background/Objectives: Antimicrobial stewardship (AMS) interventions are critical to reducing inappropriate antibiotic prescribing for acute respiratory infections (ARIs) in primary care and mitigating antimicrobial resistance (AMR). While interventions are routinely employed in hospitals, implementation in general practice is nascent. This qualitative study, part of the OPTIMAS-GP project, explored determinants influencing the implementation of evidence-based AMS strategies in Australian general practice. Methods: Using Experience-Based Co-Design, three rounds of online focus groups were conducted with ten healthcare professionals (GPs, pharmacists, microbiologist, practice staff) and ten adult patients who had experienced ARI management in primary care. Participants discussed the feasibility and acceptability of AMS interventions: shared decision-making (SDM) tools, delayed prescribing (DP) and point-of-care testing (PoCT) for C-reactive protein (CRP). Results: Thematic analysis of focus group transcriptions identified four interrelated themes: ‘Patient acceptance and engagement’, ‘Practising within a system’, ‘Prescribing stewardship’, and ‘Diagnostic stewardship’. Patient engagement was dependent upon expectations, trust, and personalised care, while systemic factors such as continuity of care, practice culture, and resource availability influenced implementation. DP was viewed as a pragmatic but potentially confusing strategy, requiring clear patient guidance and interprofessional collaboration. SDM tools were conceptually supported but challenged by time constraints and poor health literacy. PoCT-CRP was cautiously welcomed for selective use, with concerns expressed about workflow integration and overreliance on testing. Findings were mapped to the Capability, Opportunity, Motivation-Behaviour (COM-B) and Theoretical Domains Framework (TDF) to identify behavioural determinants and inform future implementation strategies. Recommendations include co-designing patient-centred AMS tools with clear instructions and red flags, enhancing GP-pharmacist collaboration, and addressing barriers to PoCT integration. Conclusions: These insights highlight the complexity of implementing AMS interventions in general practice and underscore the need for tailored, system-supported approaches to optimise antibiotic use and reduce AMR.

## 1. Introduction

In 2022, Australia ranked in the middle of 33 European countries with regard to community use of antimicrobials [1]. Although the rates of antimicrobials dispensed in Australia have been declining since 2015, the defined daily doses (the assumed maintenance doses per day according to the WHO Collaborating Centre for Drug Statistics Methodology) [2] in 2022 of 16.8 per 100 people per day were still higher than those of countries with similar healthcare systems, such as England, and more than double those reported for the Netherlands [1]. In Australia [3] and internationally [4], prescribing rates have been shown to be driven by prescribing for predominantly viral acute respiratory tract infections (ARIs) such as acute bronchitis, acute pharyngitis, rhinosinusitis, and the ‘common cold’ [3,4].

Globally, antimicrobial resistance (AMR) is increasing [5]. The misuse and overuse of antimicrobials is one of the main drivers in the development of drug-resistant pathogens [6], and prescribing for ARIs is identified as contributory [4]. The majority of antimicrobial prescribing in Australia occurs in primary care. Nationally, most prescriptions for ARIs in the community (87.3% in 2023) are provided by General Practitioners (GPs) [1]. To optimise appropriate prescribing and contain AMR, incorporating antimicrobial stewardship (AMS) interventions into primary care, and promoting AMS knowledge and skills as key competencies for all clinicians able to prescribe within their scope of practice, is advocated [1,7]. General practice, therefore, has a crucial role in reducing antimicrobial use and limiting AMR in the community [8].

Although activities to promote AMS have been supported in Australian hospitals for some time [9], interventions to enhance the quality use of antimicrobials in primary care require a different focus [7]. Effective and sustained engagement of patients within the biopsychosocial context of general practice is essential to the uptake of key messages [10], particularly for AMS [7]. Several evidence-based interventions are proposed to engage patients in AMS, such as providing antibiotic prescriptions to be dispensed later with an expectation that symptoms may resolve, known as ‘delayed prescribing’ [11], advising on symptomatic management [7], shared decision-making [8,12,13] particularly utilising decision-aids [14], and individual patient as well as general public education [15,16]. Utilising point-of-care testing (PoCT) for indicators of infection, such as C-reactive protein (CRP), is also an intervention with evidence of providing clinical decision-support and reducing antibiotic prescribing [17]. The uptake of these interventions needs to be supported by context-appropriate implementation strategies such as ongoing education and training of prescribers and audit and feedback [7,18]. However, evidence suggests that barriers exist to the successful implementation of these and other AMS interventions [7,14,19,20].

Overcoming barriers to AMS implementation requires evidence-based strategies, including active collaboration with end-users of care. Partnering with patients, their carers and families, and healthcare providers through co-design approaches has been shown to enhance users’ experiences, and the quality and safety of care [21,22]. A systematic review of 65 studies with a focus on ‘healthcare co-production’ suggested that ‘co-design’ encouraged shared goals and improved service user/provider relationships, communication, outcomes, and satisfaction [23]. ‘Experience-Based Co-Design’ (EBCD) provides a methodology that brings service providers and consumers together in a collaborative change process, or ‘co-design’ [22]. Essential steps in co-design processes are gathering and understanding the experiences of consumers and service providers, to inform measures to improve the experience [22]. EBCD can be integrated into developing implementation strategies that are supported by behaviour change theory [24].

The Optimal Implementation of Antimicrobial Stewardship in General Practice (OPTIMAS-GP) is a five-year study to investigate optimal implementation strategies to enhance the use of AMS interventions in general practice. For its first stage, a qualitative study was undertaken to inform AMS implementation in an Australian general practice setting. Using the EBCD collaborative change process [22], the aims of this qualitative study were to investigate the determinants influencing the implementation of evidence-based AMS interventions for adult ARIs in Australian general practice, from perspectives of healthcare professionals (HCPs) and patients. The results from this investigation will be used to inform later phases of the full trial.

## 2. Results

Ten consumers (six female), all of whom identified as patients, aged from 28 to 84 years and residents of metropolitan New South Wales (NSW), participated. Ten HCPs (six female), predominantly GPs, with up to 40 years of practice in NSW or Tasmanian metropolitan, regional or rural settings, also participated (See Section 4). 

### 2.1. Qualitative Analysis

**Figure 1:**
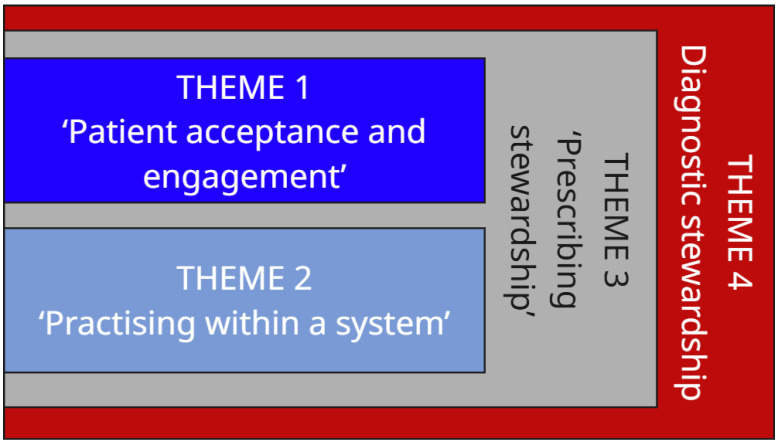
Determinants of implementation of AMS interventions: interrelated themes identified.

Determinants influencing the implementation of AMS interventions were arranged into four themes: ‘Patient acceptance and engagement’, ‘Practising within a system’, ‘Prescribing stewardship’, and ‘Diagnostic stewardship’. The latter two themes were cross-cutting, with the interrelationship depicted in Figure 1. Subthemes within each theme and determinants for implementation according to these were identified (Table 1). The following section elaborates on the themes, referring to the AMS interventions used to prompt discussions (see Table 2, Section 4.4, for specific interventions). Further supporting quotations are provided in the associated Supplementary Tables.

#### Themes

- Theme 1: ‘Patient engagement and acceptance’

This theme includes four subthemes: Patient expectations, Therapeutic alliance (personalised planning, validation, reassurance and symptomatic management), Utility and accessibility of shared decision-making patient resources and Harm minimisation, with each of the subthemes contributing further evidence. The theme explores how patients interact with AMS interventions, shaped by their expectations, the therapeutic relationship with the HCPs, the accessibility of SDM tools and strategies for harm minimisation. Overall, a dynamic interaction was described between patient belief systems, HCP communication strategies, and system constraints.

1. Patient expectations

According to our HCPs and patient participants, patient engagement and acceptance are determined by pre-existing patient expectations and how these are managed. Engagement is dependent on patients’ acceptance, or openness to education about the nature of ARIs, their expectations about receiving antibiotics, and ARI management in a post-pandemic era. Dissonant contributions to this subtheme were accounts of patients voicing opinions, their prior experiences and long-held beliefs, and how these are retained in the face of contrary evidence. HCPs sensed pressures, some related to patient circumstances, for instance, work or family responsibilities, though they acknowledged their perceptions were not always accurate. *‘[W]e’ve just got to make sure we’re not making the assumption that the agenda is antibiotics—and delve a little bit more into what other things may be impacting, whether it’s childcare, their job, family members, and whatever else that might make them feel they should have an antibiotic just in case.’ (GP2)*.

HCPs and patients reported patient pushback against hearing explanations, and the time taken in providing these, or in employing supporting resources such as the shared decision-making tool used to promote discussion. However, having recurrent and consistent discussions about the limitations of antibiotics, the role of the body’s immune system, and “busting” common myths, such as discoloured phlegm indicating a bacterial infection, were all recounted as tactics to manage expectations and engage patients. One GP reflected: *‘I’ve probably spent many, many years educating patients. So, they often go, “Oh, yeah, I thought you’d say that”’ (GP1)*. HCPs also described implicit shared decision-making that they undertook in discussions with patients, rather than reliance on a specific resource. *‘I often say…“[Y]ou’ve got this lousy sore throat, isn’t that horrible?” And then you ask them some other questions…“have you lost your voice? All that makes it very much a virus, doesn’t it?” And they go, “yes.” And you say, “oh, and you’ve got a runny nose, so it’s not just your throat…”…it’s far more powerful if you can tell the story…and take them on that journey of what’s actually happening for them.’ (GP2)*.

There was mixed feedback about managing ARIs in the post-pandemic era, with patients either reverting to their earlier beliefs that all ARIs require antibiotics or being comfortable with the concept of viruses not responding to antibiotics: *‘I wonder if COVID changed perspectives…I think it did in that people acknowledge that viruses can cause a lot of harm.’ (MB)*.

Subtheme determinants are provided in Table 1 and further supporting quotations in Supplementary Table S1.

2. Therapeutic alliance: validation, personalised planning, and symptomatic management

Achieving a therapeutic alliance with patients was recognised as a determinant of patient acceptance of AMS interventions. Several pivotal factors were identified to achieve this, for instance, HCPs establishing relationships and rapport with patients, building trust, involving patients in decision-making, and actively scheduling follow-up opportunities. For the patients, it was believing that their experiences, intelligence, and needs (illness and socio-economic) had been validated: *‘I like to be acknowledged as relatively intelligent. And I don’t want them to say, “don’t worry about that; I’m the doctor”…I want a bit of human interaction which will help me understand it.’ (PT1)*.

Individualising treatment was viewed as a determinant of uptake of AMS interventions by HCPs, to validate patients’ experiences. In their opinions, scheduling follow-up reassessments alleviates patients’ concerns and provides a safety net. *‘Since COVID happened, people are a lot more comfortable using the telephone as a way of following up…I have found that quite a valuable thing to say, well—“I’ll put your name on my list here at say 3 o’clock tomorrow”’ (GP6)* Contextually, Telehealth appointments were therefore viewed both as enabling AMS or conversely, constraining AMS interventions, due to the inability to perform a physical examination impacting on GPs’ clinical decision-making and an informed diagnosis.

Time constraints were cited as restricting personalised planning, equally recounted by patients and HCPs. ‘(GPs are) very much constrained by time…they get you in, go through the list…it can be very frustrating because they can’t expand and dig a little deeper.’ (PT5). However, GPs were aware of the value in personalising their approach for the uptake of their messages, particularly for symptom management: ‘[If] you’re able to handwrite something and the patient feels more held and validated -… that this is a personalised plan for them to get better. That said, it’s time-consuming and my writing is rubbish.’ (GP4). As a solution, HCPs perceived a symptomatic treatment plan that is able to be personalised within the consultation timeframe, similar to that easily available previously through the national QUM organisation (NPS MedicineWise) [25], as an efficient AMS intervention resource.

Subtheme determinants are provided in Table 1 and further supporting quotations in Supplementary Table S1.

3. Utility and accessibility of shared decision-making resources

Both HCPs and patients expressed doubts about the suitability of the scrutinised AMS intervention resources in supporting meaningful shared decision-making (SDM). *‘[T]he problem is, we show them statistics…if there’s 3% of people that’s going to benefit from [antibiotics], then they’re going to think they’re in that 3%. So, I’m not sure how much it would sway people.’ (GP4)* Although the purported role of the resources is to share decision-making, there was an unease perceived among HCPs and patients generally about the appropriateness of this intervention for ARI management. GPs expressed doubts about the patient’s ability: *‘So if you’re involving them with the decision-making and they make the wrong decision; then you’re kind of fixed…it’s tricky.’ (GP3)*. One GP reflected *‘[For the decision-aid to be useful]…first of all, the clinician would have to really recognise where their stance is early. So, if you were sitting at the 75 to 90% sure that they don’t need antibiotics, then you might not engage with this process and just tell them like, “look, no, suck it up”. But if you were really on the 50% and you were comfortable coming to the conclusion of prescribing, then this process could work. It’s tricky.’ (GP4)*.

Participants considered the AMS intervention resources lacked balance, that a high level of health literacy was required, and questioned their utility in the context of an acute consultation, particularly given the cognitive commitment *(‘If [people are] sick, they’re not going to feel like wading through all that.’[PT5])* and time commitment required: *‘In a busy consultation, finding [the decision-aid], bringing it up on your screen, pointing to it, it’s not that easy. It takes a lot more time out of your consultation, and it actually takes a focus away from the patient.’ (GP1)*. Interpretation of the SDM resource by the GPs was thought to be needed, although even this would not be straightforward: *‘[T]he ‘dot plot’ thing…It takes me ages to figure out what it’s trying to say. And even then, I’m just pretending to understand statistics. ….and then you just lose track of where you were and you’re like, “Wait, is this upside down somehow?”’ (GP4)* The resources reviewed were not routinely accessed, as only one HCP participant had previously viewed the Australian SDM tools [26] and that was in a research setting. Despite these observations, participants noted excerpts from the resources that might be beneficial for patient engagement, to facilitate personalised planning and support symptomatic management, although there was consensus that significant adaptation was required.

Subtheme determinants are provided in Table 1 and further supporting quotations in Supplementary Table S1.

4. Harm minimisation

The participant data contributing to this subtheme were prompted by reviewing all the AMS intervention resources. Although HCPs were committed to minimising inappropriate antibiotic prescribing, using the concept of widespread antimicrobial resistance as a rationale for limiting antibiotics was viewed as counterproductive. Patients who are acutely unwell were noted by both HCPs and patients to be less willing to consider societal or personal consequences: *‘I think that the altruism when you’re feeling really sick, often runs low, so not taking antibiotics ‘cause you think you’re going to save the planet is probably not a big motivator for most people…telling them they’re doing a good thing when they’re feeling like crap….they just want to get better.’ (GP1)’* There was a range of awareness of the concept of antimicrobial resistance within our patient cohort, or its implications: *[W]e hear about antibiotic resistance and it’s …like, “oh, it might happen”…I don’t really have an understanding about antibiotic resistance…The benefits outweigh the risks…It’s a risk, but whatever!’ (PT7)*.

However, both HCP and patient participants recalled how focusing on possible personal harm (antibiotic-related side effects such as thrush, diarrhoea, individual resistance) and economic cost (prescription and side effect treatment costs) had, or could, improve the uptake of interventions. In this context, follow-up Telehealth appointments were promoted as a practical AMS intervention (aligning with the preceding subthemes), in reassuring patients, managing their expectations, and minimising the possible harm and costs from inappropriate antibiotic prescribing. Additionally, scheduling a follow-up appointment if possible was preferred over providing a prescription with instructions for delayed dispensing.

Subtheme determinants are provided in Table 1 and further supporting quotations in Supplementary Table S1.

- Theme 2: ‘Practising within a system’

Implementation of AMS interventions was viewed as dependent upon the individuals practising within a system, and the influence of the system on the patient–prescriber interaction, which comprises the two subthemes for this overarching theme. This theme offers perceptions of how implementation of AMS interventions can be shaped by the individual’s clinical judgement, but also by the broader healthcare system.

1. Individual practitioners

The clinical acumen of clinicians was identified by HCPs as a determinant of implementing AMS interventions and, indirectly, by patients. HCPs reflected on the utility of AMS guidelines and resources in their practice and in patient interactions. They also deliberated on the practices of less-experienced or AMS-cognisant colleagues: *‘[The patient says] “I always need these”…really? have we given you the opportunity to see if you actually do? actually, “it’s not that you’ve needed them, it’s that the doctors always prescribed them. There are no alternatives that you are aware of”.’ (GP5)*. HCPs referred to their own limited knowledge of resources to promote AMS, such as ‘delayed prescribing’ and symptomatic management templates: *‘I do [advise on symptomatic management]. I write it all by hand. So that makes me feel a bit of an idiot, that I haven’t found a proforma for this. ’(GP5)*.

Our HCPs reflected on recent dilemmas in acute ARI management; for instance, their awareness of contemporary peaks in pneumonia and their fear of consequences of missing serious diagnoses, while simultaneously aiming to implement AMS interventions.

When presented with the AMS intervention of PoCT of CRP in the general practice, individual HCPs voiced both scepticism and curiosity about its utility in assisting in ARI management and uncertainty around the evidence base: *‘I was…super interested on the evidence behind it…, but I think we’re all pretty sceptical’ (GP3)*. A single GP was keen to observe its role in ARI management: *‘Well, I can see this becoming an important tool. I love it! (GP6)’*.

The HCPs expressed a sense of loss of clinical acumen and questioned the additional benefits to patient management. However, there was consensus on the potential benefits and reassurance obtained from negative results to provide objective evidence to support management decisions and reduce inappropriate antibiotic prescribing. GPs agreed they would be selective in the patients they would test.

Subtheme determinants are provided in Table 1 and further supporting quotations in Supplementary Table S2.

2. System influences on patient–prescriber interaction

A lack of continuity of care, variations in practice that patients receive and the inherent mixed messages conveyed, and the paucity of urgent or ‘book-on day’ appointments were all surmised as deterring therapeutic alliances, patient engagement, and subsequently, the uptake of AMS interventions. Both HCPs and patient participants noted variation in ARI management, depending on the GP or pharmacist consulted, as well as the days of the week or times of day. *‘[I]n the last 12 months it’s been difficult because even though I’ve remained in the same practice, I’ve had a lot of doctors who have been doing their residency there and then have moved on. So, I haven’t had that continuity of care…the information I get is slightly different each time.’ (PT7)* HCPs recalled patients actively seeking other opinions if a trusting relationship had not been established or if their usual GP was unavailable, contributing to concerns voiced about HCPs reinforcing outdated messages. They related second opinions being sought after-hours, frequently via the Australian federally funded ‘Urgent Care Centres’, implemented to reduce pressures on GPs and emergency departments. While these services provided accessible care, participants reported that patients had received differing perspectives, requiring GPs to spend additional time reconciling these during subsequent consultations. However, if an initial ARI consultation had been possible, scheduling follow-up appointments for review, particularly using Telehealth, was noted as enabling continuity of care.

Delays in adopting changing practices, particularly at a practice level, were viewed as determining factors in the implementation of AMS interventions. Participants suggested that these led to variations in clinical practice. Strategies proposed to counter this were engendering a practice culture to promote peer-to-peer support, normalising audit and feedback, and providing training and supervision. *‘[Registrars] get lots of education…they’re stepping through these cases, and you are either seeing the patient with them, or you are having a sit-down discussion either at the time or we do what we call ‘random case analysis’. [A GP] in our practice…used the NPS respiratory infection audits pretty much every winter…Which were really good because it made you reflect on what you did, and it then became set in your mind of how you should do it and what were your criteria.’ (GP02)*. The recurring strategy suggested by HCPs, particularly GPs, was the utility of electronic current therapeutic guidelines and resources to confirm their diagnoses, and to share with patients in support of their decisions. Consequently, best practice was viewed as threatened if these strategies and resources were not readily available at a practice level. Additional strategies to support the implementation of a practice-wide AMS approach, suggested by HCPs, were to remove obsolete antibiotic prescriptions from patients’ current medical records and default repeats in prescribing software.

Regarding PoCT of CRP, GPs expressed concern about the challenges of incorporating this intervention into practice. Alternatively, with previously established efficient workflows, CRP testing was viewed as comparable to that of other routine tests, such as urine or blood sugar analysis, and could be incorporated into patient consultations: *‘[I]f you know it’s going to happen, you can almost plan for it beforehand. If I’m seeing a patient, and I’m thinking, I want to do a BSL for you today, I am going to word that up from the start. And we’re going to be working on getting you in the right frame of mind as well, for young people, it’s not a pain-free procedure so it’s something that you have to warn them about that aspect. It’s a bit different to the older patients that do this stuff all the time.’ (GP5)* Another concern raised was the cost of an additional test, who would be responsible, and whether these would be outweighed by any realised benefits. *‘But how much are the CRPs? …if we’re going to use this long term…we charge for pregnancy tests, we charge for everything…But what about the CRP sticks? Are they going to cost a heap?’ (GP4)*.

There was little patient input into this subtheme, except for their interest in the availability of this in Australia, and potential acceptance of its use in practice: *‘Well, I’d say [my doctor] was being thorough and it’s eliminating any question.’ (P10)*.

Subtheme determinants are provided in Table 1 and further supporting quotations in Supplementary Table S2.

- Theme 3: ‘Prescribing stewardship’

This theme has relevance to the two themes previously described. It incorporates three subthemes: ‘Delayed prescribing’ as a concept—a strategy to advance AMS—and ‘Delayed prescribing’ as an AMS intervention or tool—to foster patient engagement. A third subtheme identified was Interprofessional collaboration—recognised as both required and enhanced by this AMS intervention. There was consensus among participants that a systems approach to ‘delayed prescribing’ was mandatory for it to be a successful AMS intervention, that is, adopting a stewardship approach to prescribing. This theme explores ethical, communicative, and systemic considerations of delayed prescribing. It is a contested yet potentially innovative AMS intervention. It describes tension between clinical pragmatism and patient perception, and the necessity for collaborative clarity across healthcare roles as well as systems support.

1. ‘Delayed prescribing’ as a concept

Although HCPs, particularly GPs, acknowledged the pragmatic role of ‘delayed prescribing’ in harm minimisation—to reduce the community antibiotic load—providing a prescription on the proviso that it be dispensed only if symptoms worsen raised concerns about its implementation. GPs viewed the use of ‘delayed prescribing’ as providing mixed messages…*‘[T]his just might be my own discomfort—we have told a patient, “You don’t need antibiotics, but actually here’s the script if you do.” It signals a little bit of, “I don’t really know. You probably do”.’ (GP1).* Some patent feedback echoed this sentiment, with irritation: *‘[T]he very nature of…‘delayed prescribing’ is like, “you mean I can have it, but I just want to wait a bit longer”. Then it’s really testing your patience.’ (PT2)*: Other patient feedback was dissonant, ranging from reassurance to perplexity: *‘But what is the point of ‘delayed prescribing’ anyway, please? …I’m not talking about the process; I’m talking about the philosophy behind it.’ (PT1)*.

The acceptance of Telehealth for follow-up, enabled during the pandemic, was preferred as a method to enhance patient engagement and was preferred to providing a standalone ‘delayed’ prescription. *‘[I]f I can do the quick catch up down the track, I like that better…But there are so many situations where you can’t do that. There’s the patients who are travelling, there’s the weekend or the last day before your holidays…they seem to happen a lot more often than I would like.’ (GP2)*.

Subtheme determinants are provided in Table 1 and further supporting quotations in Supplementary Table S3.

2. ‘Delayed prescribing’ as an AMS intervention

As emphasised by one GP, the practice is more correctly delayed *dispensing*, as prescribing is relatively immediate. However, HCPs shared accounts of the prescriptions being dispensed without delay and either used immediately or kept ‘just in case’, and of sharing with family and acquaintances, and that a level of trust was assumed to minimise this. The patient perspective was confusion or uncertainty of the trigger for having the prescription dispensed in the absence of specific instructions*: ‘[My doctor] said, “I’ll give you this and see how you feel. If you think you need it, you can go and get the prescription.’’…I wasn’t too sure what he meant, at what point or what should I feel…what should trigger me going to get the antibiotics?’ (PT1)*. Patients also related experiences of taking the antibiotic at a later time, but for a different reason. To reduce the likelihood of immediate dispensing, one proposed approach was to provide specific rather than vague details about when the antibiotic *may* be indicated, and to augment the ‘delayed prescription’ with advice about how to manage their illness rather than relying on the antibiotic. *‘[Instead of ‘delayed prescribing’], use the word ‘if this happens’ or ‘if you’re still feeling horrible or worse, then do such and such’…that might be clearer.’ (PT5)*.

Subtheme determinants are provided in Table 1 and further supporting quotations in Supplementary Table S3.

3. Interprofessional communication

Limited interprofessional communication was regarded by HCPs as threatening the success of ‘delayed prescribing’ as an AMS intervention. However, utilising prescriptions as a communication tool for their pharmacist colleagues, by including a prescription expiry date, was proposed by GPs as supporting their intentions to limit the currency of the prescription, to promote conversations. Pharmacists echoed this approach: *‘[W]hen we see a script, if it says something like, “do not…dispense before this day or after this day”… That’s really helpful for delayed prescribing.’ (PC1)* The additional pharmacist role described was seeking clarification of GPs’ intentions before dispensing, particularly for old prescriptions, and encouraging their colleagues to adopt this practice: *‘I will not fill [old antibiotic scripts] unless I’ve had a conversation with [the patient]. And I strongly discourage them unless they’re on a regime…to self-initiate…[if] they’ve got clear guidelines, I’m happy with that. And, if they’re very adamant, I will be happy to call the doctor for them.’ (PC1)*.

Subtheme determinants are provided in Table 1 and further supporting quotations in Supplementary Table S3.

- Theme 4: ‘Diagnostic stewardship’

This final theme integrates elements from the three preceding themes, with subthemes identified: Clinical acumen and decision-making, Overreliance on testing, Result interpretation, and Patient engagement and acceptance. The theme describes the balance required between clinical judgement and diagnostic testing, emphasising the need for selective use of diagnostics to support AMS. It considers the challenges encountered between technological reliance and professional expertise, and their impact on both clinician behaviour and patient engagement.

1. Clinical acumen and decision-making

HCPs identified clinical acumen and skill in differential diagnosis, both viewed as products of experience, as advancing AMS. Confidence in their clinical decisions and ability to *not* prescribe *or* order excessive diagnostic tests were observed as significant. The expertise of clinicians was recognised as aligned with their clinical decision-making. Additionally, some HCPs questioned if additional tests, such as PoCT of CRP, would augment clinical decision-making, despite this being the rationale for this AMS intervention. There was a possible role perceived for additional testing if the clinical examination was equivocal: *‘[M]y idea for the utility for the CRP would be if I was very much on the fence…If I had someone who I wasn’t going to prescribe for and then we did the CRP and it came back positive, then I’d have to decide, okay, do I actually want to give you antibiotics?’ (GP4)* Consequently, the consensus preference was for the *selective* use of additional diagnostic tests, specifically the CRP, for a patient with an ARI.

Subtheme determinants are provided in Table 1 and further supporting quotations in Supplementary Table S4.

2. Overreliance on testing

GPs expressed hesitancy in employing additional diagnostics within general practice, in the context of the availability of PoCT of CRP. *‘[L]ooking at that flow chart [for PoCT of CRP] it first struck me as, gee, we’re doing a lot of tests. It was [previously] a clinical decision…Now we’ve got the COVID test, influenza, maybe RSV added to that… and then we do a CRP. It feels a little like again, we are relying on tests and not using our clinical judgement nearly as much.’ (GP1)* Additionally, with increased reliance on testing, there was concern from the HCPs of the potential for loss of clinical acumen and for hindered skills-acquisition in early career practitioners. ‘*I’m a strong advocate for diagnostic stewardship. So, I wouldn’t encourage further testing…testing leads to testing leads to testing’ (MB)*.

Subtheme determinants are provided in Table 1 and further supporting quotations in Supplementary Table S4.

3. Result interpretation

Concerns were expressed of possibly missing the CRP peak and cases of serious sepsis, reinforcing the hesitancy of relying on a single test and emphasising that follow-up planning is still required. *‘[A low result] maybe gives false reassurance that this couldn’t develop into something else*. *And the problem with CRP…We know that it lags and can take up to 72 h to be demonstrating its peak…if we rely on it too heavily…we miss cases of serious sepsis. You wouldn’t expect to miss it with clinical acumen. But that if you are saying it’s the be-all and end-all test, then that’s the risk.’ (MB)* There were also concerns about the non-specific nature of CRP test results.

Subtheme determinants are provided in Table 1 and further supporting quotations in Supplementary Table S4.

4. Patient engagement and acceptance

Our HCPs reflected that post-pandemic, many of their patients prefer the physicality of being seen and examined. There was some agreement that, if selectively used, PoCT of CRP had the potential to enhance patient engagement and acceptance when results were negative (contributing to our first identified theme). The conflicting perspective was that in many cases, the expertise of the GP should be sufficient: *‘If you 100% think no, this person does not need an antibiotic, then you technically should just be putting your foot down’. (GP4)*.

Subtheme determinants are provided in Table 1 and further supporting quotations in Supplementary Table S4.

## 3. Discussion

The findings from our investigation into determinants influencing the implementation of evidence-based AMS interventions for adult ARIs were able to be assigned to four overarching themes: ‘Patient acceptance and engagement’, ‘Practising within a system’, ‘Prescribing stewardship’, and ‘Diagnostic stewardship’. The themes and their subthemes were interrelated and interdependent; for instance, selective testing of PoCT of CRP was viewed as a method to engage the patient, deliver an objective measure for a diagnostic dilemma, while keeping within the boundaries of diagnostic stewardship. Determinants characterised in themes were those at the patient level (for example, their beliefs and expectations, acceptance of education, loss of altruism and health literacy), practitioner-level (for example, managing patient expectations, harm minimisation, pressures on GPs, clinical acumen and selective use of diagnostics) and systems level (for example ‘Delayed prescribing’, Telehealth, PoCT-CRP, interprofessional collaboration). Additionally, there were determinants dependent upon the interaction of these, for example, SDM, therapeutic alliance, validation and reassurance, personalised planning, ‘Myth-busting’, and continuity of care.

Engagement of patients in interventions by inviting shared decision-making is frequently recommended in guidelines and research to support AMS [8,12,13,18]. However, both patient and practitioner participants in our study were dubious of the utility of the Shared Decision Making (SDM) tools reviewed in our focus group discussions, despite these being currently nationally recommended [18]. Although participants approved of the concept of SDM, there was doubt about its applicability in acute ARI presentations, when unwell people are less likely to engage. While a 2015 Cochrane meta-analysis of data from nine randomised controlled trials with nearly 500,000 patients found that using SDM as an AMS intervention significantly reduced antibiotic prescribing for ARIs in primary care [3], barriers to successful implementation remain. For example, a recent Australian study in general practice settings reported no differences in antibiotic prescribing for ARIs between SDM-intervention and control groups [27]. Analysis of follow-up survey data from this latter study found that while SDM-intervention group GPs had access to a 15 min training video, not all undertook the training, nor used the SDM tools. One of the most frequent reasons given for not using the aids was that patients found the SDM tool content difficult to understand [27], which aligns with our findings. One approach to improving patient acceptance and engagement with AMS interventions proposed by our participants was providing reassurance to people with an ARI and validating their illness, as a means of engendering trust and strengthening the therapeutic alliance. These observations are not new and echo much of the AMS literature [20,28,29,30,31] and illustrate that co-design of a patient-friendly resource is required. The outcome from similar research to ours, although for the management of urinary tract infections, is a suite of co-designed resources [32].

The themes of ‘Practising within a system’ and ‘Prescribing stewardship’ were strongly linked when considering participants’ feedback on ‘delayed prescribing’ (DP) as an AMS intervention, which drew disparate responses from our participants. Although some HCPs disagreed with the inherent messages of DP, there was a concession to the practice as a recourse when other strategies were not possible. Patient responses were similarly dissonant. This finding supports a comparable study from Switzerland, which found polarised responses to DP around attitudes, convenience, expectations of autonomy, and the influence and effect on the patient–doctor relationship [30]. Notably, in that study, one participant voiced their disagreement: *‘what does it even mean “if the symptoms worsen”?’* echoing sentiments expressed by our participants. Our participants agreed that the success of DP relied on interprofessional collaboration between GPs and pharmacists, as well as system changes, potentially employing technological solutions to enable expiries embedded into prescriptions. Indeed, supporting system-wide adoption was proposed as a prime determinant to implementing AMS interventions, with specific mention given to audit, feedback and education, addressing both the ‘Practising within a system’ and ‘Prescribing stewardship’ themes. This latter approach is supported by research calling for audit and feedback at a practice level to be integrated into healthcare systems to reduce inappropriate antimicrobial prescribing and AMR [33,34].

The cross-cutting theme of ‘Diagnostic stewardship’ is pertinent to the preceding themes. Resolute participant feedback from our study was that use of diagnostic tests, in this case PoCT of CRP, is not an AMS intervention to be implemented in isolation. A systematic review and meta-analysis of 13 randomised controlled trials by Martinez-Gonzales et al. [35] concluded that CRP testing significantly reduced immediate antibiotic prescribing compared with usual care. However, at 30 days, the review found this was not sustained, and there were significantly higher rates of re-consultation [35]. A recent systematic review and qualitative synthesis of studies (*n* = 33) of primary healthcare professionals’ and patients’ views investigated factors affecting PoCT uptake, primarily of CRP [36]. HCP factors similar to those from our findings were identified, although further categorised into three ‘high-level’ sets. These included the following: those that occur outside (HCP doubt and systems or workflow concerns), within (scenarios where PoCT adds value, influence on decision-making, interpretation of results and patient engagement), or after (patients will demand future testing) a GP consultation. Interventions were identified to support the use of PoCT, including training HCPs in its use, optimising technology, setting up a framework to support PoCT quality, including PoCT in clinical guidelines, training HCPs in result interpretation and communication skills [36]. Further research into these proposed interventions is warranted. Our only patient input into the use of CRP was echoed among the patient perspectives of this study, and that was trusting in their HCP’s ability.

With our study set in the post-pandemic period, the finding of an impact of Telehealth is a recent consideration in primary care AMS. Our patient cohort expressed dissonance as to their consultation preferences. However, our HCPs were united in their frustrations with physical distancing in restricting meaningful investigations, but also an appreciation of the place for Telehealth for patient follow-up, to allay concerns, and provide reassurance. There have been mixed results in Australian research on the effect of Telehealth compared to face-to-face consultations on the provision of antibiotic prescriptions, with both increases [37] and reductions determined [38], although the benefits as proposed by our HCPs were not investigated in these studies.

There are few comparative studies of understanding impediments to implementing AMS interventions in primary care, from perspectives of both HCPs and patients. Jeffs et al. gathered perspectives from GPs and clinic team members while they participated in a multi-site AMS programme [39]. Some of the AMS interventions, such as ‘Delayed antimicrobial prescriptions,’ were similar to those discussed with our participants. There was no patient voice in this research; however, interviewed HCPs (*n* = 23) recognised the practical utility of DP at times when patient follow-up was not assured. Also, that patient engagement and acceptance of messages were crucial for uptake of interventions, with time constraints and patient expectations limiting these. Concordant with our findings, systems-level determinants were reflected as influencing implementation, such as assuring continuity of care.

A combination of interventions is key to AMS implementation. Interviews of clinician participants in a multi-site European study, which included the use of PoCT of CRP and training in patient communication and resources for patient education, were undertaken to explore their perspectives [40]. Interestingly, the findings regarding PoCT of CRP reflected the ambivalence voiced by our HCPs: time constraints, difficulty in incorporating into practice, and concerns about over-testing, but also their selectivity in using the test, and the benefits in diagnostic uncertainty and for patient engagement [40].

### 3.1. Strengths and Limitations

In developing themes, this study benefitted from the triangulation of qualitative data from both HCPs with broad experiences as well as from adult patients of varying ages. Recruitment was pragmatic, using existing networks, with attempts made to broaden recruitment and improve diversity. Our HCPs were found to be representative of differing socio-economic and geographical locations. All who expressed an interest were included as participants. It was apparent that our HCPs were aware of AMR and the necessity for AMS interventions. Their perspectives on interventions were not always unanimous; however, their prior knowledge potentially influenced our findings. Their wide range of experiences and interactions with less-inspired medical and pharmacy colleagues were also shared during the focus group discussions and incorporated into our findings. All our patient participants were able to share insights from their own experience of having an ARI and seeking management from HCPs. They were all current residents of metropolitan NSW, but were diverse in age, background, and literacy. A limitation of our study was the limited heterogeneity of the patient group in comparison to the diverse communities seen in Australian primary care. This was particularly relevant for the patient group, drawn entirely from metropolitan regions. As a result, the conclusions drawn from this sample may not represent the full diversity of views in Australian general practice. It is possible that having an even more varied patient group and in greater numbers would alter some of our findings, given the heterogeneous nature of Australian primary care. An additional limitation was the inclusion of only one infectious disease specialist, which may limit the analysis. Future work should include a range of non-GP medical specialists who support AMS in primary care.

The methodological approach employed—three successive focus group sessions per participant group—generated in-depth and lively discussions, with iterative input, and allowed our research team to remain close to the data. This method assisted in employing EBCD principles in our research. Our findings closely reflect the language used by participants. We acknowledge, however, that they are context- and location-relevant, and may be less applicable to different healthcare systems.

### 3.2. Practical Implications and Future Areas of Research

Further research is required to address the implementation of AMS interventions in primary care. To ensure high patient engagement, AMS interventions are essential that offer personalised instructions about non-antibiotic, symptomatic management, but importantly provide information on red flags and triggers to having an antibiotic dispensed, if using DP. Co-design with patients is required to ensure such tools are concise, clear, address different levels of health literacy, and yet are adequately informative. Practical advice from our findings is that combining AMS interventions and strategies has potential for greater impact. Recently co-designed Australian information leaflets, in early stages of implementation, are useful to provide essential information about ARIs in a patient-friendly format [41]. Our participants, however, advocated embedding mechanisms to support personalised and specific treatment planning, along with relevant information. Ongoing research is furthering investigations into the utility of our co-designed treatment plans, used in addition to DP and PoCT of CRP as appropriate [42]. Telehealth is a system-level AMS intervention requiring greater funding and support [43], and an investigation into its utility as an alternative to DP, to allay patient concerns and provide reassurances. There is also a clear benefit in building interprofessional collaboration between GPs and pharmacists to strengthen system-level support and prescribing stewardship. Findings of ongoing relevant Australian research into such collaboration will influence the optimal implementation of models of care [20,44]. Finally, while the selective use of PoCT of CRP can be used to support diagnosis stewardship, significant barriers must be addressed to ensure optimal implementation. The next stages of our OPTIMAS-GP study aim to further investigate these [42].

## 4. Materials and Methods

### 4.1. Design

A series of online focus group discussions was conducted, guided by EBCD principles [22], as part of the larger OPTIMAS-GP study investigating implementation of AMS interventions in Australian general practice. The study had approval from the University of Wollongong Health and Medical Human Research Ethics Committee (2024/015) on 5 April 2024.

### 4.2. Participants and Setting

**Figure 2:**
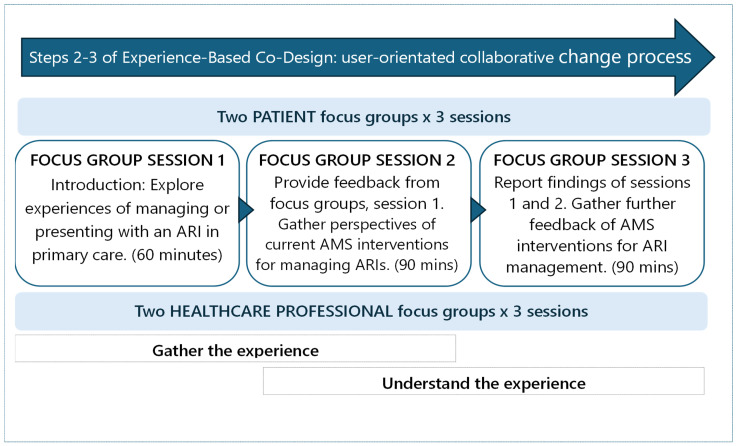
Structure and activities in healthcare professional and patient focus group discussions, guided by Experience-Based Co-Design (EBCD) principles [22]. ARI: Acute respiratory tract infection, and AMS: Antimicrobial stewardship.

Two groups of Australian HCPs (GPs, pharmacists, general practice personnel, and a clinical microbiologist who is also an infectious disease physician) were recruited from the research team’s professional networks. Two adult consumer groups (patients and/or carers in Australian general practice) who had experienced an ARI managed in primary care were also recruited through extended healthcare networks to participate in focus group sessions conducted separately from the HCPs. For each participant type, there were two groups of 5 participants (that is, 10 HCPs and 10 patients in total). All participated in the three sequential focus groups (Figure 2). In total, 12 focus groups were conducted.

### 4.3. Participant Characteristics

Details of the age, sex, years of practice, practice scopes and geographic location of HCPs, and the age, sex, and location of residence of consumers were collected during recruitment. The Australian Modified Monash (MM) Model [45] was used to categorise the geographic remoteness of locations of practice or residence.

**Table 2:** Demographics and practice details of participant healthcare professionals.

Discipline	Gender	Years in Role	Scopes of Practice *	Location *	Modified Monash (MM) Model *
**Medical Practitioner, *n* = 7**					
GPs, *n* = 6	M (3) F (3)	9–40	Researcher Supervisor (GP registrars, students) Medical educator (GPs, students) Mental health, vulnerable populations Psychiatry, paediatrics, mental health University teaching appointment Hospital practice	NSW (5) Tasmania (1)	MM 2 (regional), *n* = 1 MM 3 (large rural), *n* = 4 MM 5 (small rural), *n* = 2
Microbiologist and Infectious Diseases physician (MB), *n* = 1	F	~10	Hospital Locum Disadvantaged international health services	NSW Tasmania	MM 1 (metropolitan) MM 2
**Pharmacists (PC), *n* = 2**					
	M (1) F (1)	30–40	Community Pharmacy General Practice	NSW	MM 1
**General Practice personnel (GPP), *n* = 1**					
	F (1)	15	Senior receptionist	NSW	MM 3

* ≥1 possible, MM = Modified Monash (categorisation of geographic remoteness of locations of practice or residence) [41], NSW = New South Wales, GP = general practitioner, M = male, F = female, MB = microbiologist and infectious diseases physician, PC = Pharmacist; and GPP = General practice personnel.

### 4.4. Procedure

Focus group sessions took place between May and July 2024, were conducted online and facilitated by a member of the team (MJ), using PowerPoint presentations and Zoom videoconferencing. Examples of currently available AMS interventions were included in the PowerPoints to promote participant interaction. For the first of three sessions, a discussion guide was used to gather participant experiences of either presenting with or managing an ARI in primary care (Appendix A). The guide was developed to explore and understand participant experiences, consistent with the EBCD principles [22]. Experiences collected during the first sessions were analysed by the research team to iteratively guide the following focus group discussions. In the second round of focus group sessions for both patients and HCPs, a synopsis of similarities and differences in the experiences shared in the first sessions was provided to participants to confirm understanding. Throughout the second and third sessions, contemporary AMS interventions (detailed below) aimed at improving the management of ARIs were used as prompts and scrutinised to investigate their suitability and feasibility for implementation in general practice, and potential use in the broader AMS study (Figure 2). Discussions were recorded, de-identified, professionally transcribed verbatim, and checked for integrity by the primary author (MJ).

### 4.5. AMS Interventions

Contemporary Australian and international resources that had been designed to enable appropriate antibiotic prescribing for ARIs in primary care had three strategic purposes: to facilitate 1. Shared decision-making (SDM); 2. ‘Delayed prescribing’ (DP) of antibiotics, and 3. Clinical decision support (described in more detail in the following section). Although providing advice on symptomatic management is also a recognised AMS intervention [7], there was no specific resource used in this instance to prompt discussion among our focus group sessions.

#### 4.5.1. Shared Decision-Making (SDM) Resources

The SDM resources were decision aids currently available through the Australian Commission on Quality and Safety in Healthcare (ACSQHC) [26] for use by prescribers to facilitate conversations with adult patients presenting with sinusitis, sore throat, or acute bronchitis. The decision aids are three pages in length and provide visualisations of results from collated studies that compared symptom resolution for each condition, with or without antibiotics. Additional advice is included about potential harms and general information about antibiotics.

#### 4.5.2. ‘Delayed Prescribing’ (DP)

The ‘Delayed prescribing’ resource was from the United States Centers for Disease Control and Prevention [46], designed to be given as an adjunct to an antibiotic prescription that has been provided with the proviso that it be dispensed at a later time, and only if required. It also lists suggestions for symptom relief and general statements about possible adverse effects of antibiotics.

#### 4.5.3. Clinical Decision-Support Resources

Two types of resources were used. From the United Kingdom National Institute for Clinical Excellence (NICE), algorithms for the management of acute sore throat [47] or acute cough [48] were shared with participants. Additionally, an algorithm for the use of PoCT of CRP in adults with ARIs, from the Primary Care Respiratory Society [49], was shared with HCPs to investigate perspectives.

### 4.6. Data Analysis

Participant demographic data were collated using descriptive statistics. The focus group data were analysed using the principles of inductive thematic analysis [50] to investigate the research question, that is, to investigate the determinants of the implementation of evidence-based AMS interventions for adult ARIs in Australian general practice, from perspectives of healthcare professionals (HCPs) and patients.

All transcriptions were independently coded in NVivo by two coders (MJ and MAB), who reviewed and reread all transcripts. Codes, identified through triangulation of both HCP and patient data, were compared and consensus reached, and categorised into broader overarching themes. Themes were presented to a panel of other researchers, who familiarised themselves with a proportion of the transcripts (CC, JR, CM) and were then categorised according to the determinants of implementation by consensus, with additional input by research team members (AB, JM).

Analysis was informed by the Theoretical Domains Framework (TDF) [51,52,53]. The domains of this framework can be condensed into core components or conditions for behaviour change, which propose that Behaviour can be understood through an interaction between Capability (physical and psychological), Opportunity (social and physical), and Motivation (automatic and reflective), that is, the ‘COM-B system’ [53]. These interconnected frameworks provide guidance on the determinants of current and desired behaviours, such as antibiotic-prescribing [49], and have been used in assessing behaviour change in Australian general practice [54,55].

The identified determinants of implementation of AMS interventions were able to be mapped to the TDF [51,52,53] and COM-B [49] frameworks to identify potential targets for behaviour change strategies (Supplementary Table S5).

## 5. Conclusions

There is an urgent need to strengthen the implementation of Antimicrobial Stewardship (AMS) interventions in primary care. Our research demonstrates that improving AMS implementation in general practice requires a biopsychosocial approach that facilitates and integrates SDM, DP, and PoCT of CRP. Systematically embedding these strategies and harnessing their synergistic effects offers the potential for meaningful and sustained implementation of AMS interventions, given the scale of antibiotic prescribing in primary care.

## Figures and Tables

**Table 1:** Determinants of implementation of AMS interventions, categorised by theme and subtheme.

Theme 1 Patient Acceptance and Engagement				
**Subthemes**	Patient expectations	Therapeutic alliance: validation, personalised planning and symptomatic management	Utility and accessibility of shared decision-making resources	Harm minimisation
**Determinants**	Patient beliefs Pressures on GPs Managing expectations Patient acceptance of education Shared decision-making Myth-busting	HCP and patient relationships Building trust Scheduling follow-up Shared decision-making Efficient personalised approach Validation and reassurance Symptomatic management Scheduled follow-up reassessment Telehealth and inability to physically examine Time constraints	Utility in decision-making, personalised planning, symptomatic management Time commitment Health literacy	Loss of altruism Minimising economic and medical harm Scheduling follow-up and Telehealth Delayed prescribing
**Theme 2 ‘Practising Within a System’**				
**Subtheme**	Individual practitioners		System influences on patient–prescriber interaction	
**Determinants**	Clinical acumen Outdated practices Limited knowledge of resources Diagnostic uncertainty Individual response to PoCT of CRP use in general practice		Continuity of care Variations in ARI management Scheduled follow-up appointments Telehealth for follow-up Peer-to-peer support Audit and feedback Training and supervision	
**Theme 3 ‘Prescribing Stewardship’**				
**Subtheme**	Delayed prescribing as a concept		Delayed prescribing as a tool/AMS intervention	Interprofessional collaboration
**Determinants**	Inconsistent messages Patient reassurance		Antibiotic dispensing not as intended Patient uncertainty as to trigger Adjunct patient advice	Practice (systems) approach to manage ‘delayed prescriptions’ Optimising ‘delayed prescribing’ strategy
**Theme 4 ‘Diagnostic Stewardship’**				
**Subtheme**	Clinical acumen and decision-making	Overreliance on testing	Result interpretation	Patient engagement and acceptance
**Determinants**	Confidence in clinical skills Selective use of diagnostics	Overreliance on diagnostic tests Hindrance to skills acquisition	Concern re missing diagnosis	Patient engagement

## Data Availability

The data presented in this study are available on request from the corresponding author due to ethical reasons.

## Supplementary Materials

The following supporting information can be downloaded at: https://www.mdpi.com/article/10.3390/antibiotics14090914/s1, Table S1. Theme 1: ‘Patient acceptance and engagement’–a determinant of implementation of AMS interventions in general practice, Table S2. Theme 2: ‘Practising within a system’–a determinant of implementation of AMS interventions in general practice, Table S3. Theme 3: ‘Prescribing stewardship’–a determinant of implementation of AMS interventions in general practice, Table S4. Theme 4: ‘Diagnostic stewardship’–a determinant of implementation of AMS interventions in general practice, Table S5: Determinants of implementation of AMS interventions: themes and subthemes mapped to Theoretical Domains Framework (TDF) and COM-B components (Capability, Opportunity and Motivation)

## Abbreviations

The following abbreviations are used in this manuscript:

ACSQHC	Australian Commission on Quality and Safety in Healthcare
AMR	Antimicrobial resistance
AMS	Antimicrobial stewardship
ARI	Acute respiratory tract infection
COM-B	Capability, Opportunity, Motivation-Behaviour change wheel
CRP	C-reactive protein
DP	Delayed prescribing
EBCD	Experience-Based Co-Design
GP	General Practitioner
GPP	General practice personnel
HCP	Healthcare professional
MB	Microbiologist
MM	Modified Monash
NICE	National Institute of Clinical Excellence
NSW	New South Wales
OPTIMAS-GP	Optimal Implementation of Antimicrobial Stewardship in General Practice
PC	Pharmacist
PoCT	Point-of-care testing
SDM	Shared decision-making
TDF	Theoretical Domains Framework

## Appendix A

Focus Group Discussion Guides—adapted for each focus group audience (i.e., patients or healthcare professionals).

Session 1 (60 min)

1. Welcome and introductions, including confirming permission to audio-record the session for transcription, and ability for participants to leave the session at any time. Turn on recording—10 min.
2. Briefing slide presentation and answer any questions—5 min.
3. Set scene for the context of study and use of the study materials—30 min.

Sample questions to stimulate group discussion:

I would like you to cast your mind back to the last time, or last few times, you had a consultation for a respiratory tract infection in general practice—e.g., sore throat, cough, runny nose, sinusitis (or you consulted a doctor or healthcare practitioner)

What were your expectations of the consultation?

Can you discuss what your experience was like of that encounter?

In what ways did you feel the consultation went well?

In what ways were you not happy with aspects of the consultation?

What were your expectations of the use of antibiotics to treat the infection—yours and the GP/patient (as appropriate)?

Are there things that you feel would have helped before, during or after that consultation to improve the experience?

4. Introduce study materials for participants for familiarisation before next session, offer to send copies if participants would like—10 min.
5. Wrap up and answer questions—5 min.
