# Supplementary material for: Determinants of Implementation of Antimicrobial Stewardship Interventions for Managing Community Adult Acute Respiratory Infections: Qualitative Analysis from the OPTIMAS-GP Study Co-Design Phase
Source: Antibiotics (Basel). 2025 Sep 11;14(9):914. doi: 10.3390/antibiotics14090914 (PMC12466759; doi:10.3390/antibiotics14090914)
Supplement: Supplementary file 1 [file antibiotics-14-00914-s001.zip › Supplementary Table S1.docx]

*Table S1.* *Theme 1: ‘Patient acceptance and engagement’ – a determinant of implementation of AMS interventions in general practice*

| Theme 1: PATIENT ENGAGEMENT and ACCEPTANCE |
| --- |
| Patient expectations |
| Patients expecting antibiotics  ‘*They’re all anxious, that they're going to get sicker and miss days of work….[T]hey've been coughing up green phlegm, they would expect to get antibiotics.’ (PC1)*  *‘It's the patient’s experience and their beliefs […] that is probably one of my biggest challenges when I'm trying not to prescribe antibiotics to someone.’ (GP1)*  Pressure on GPs  *‘I've seen [the NICE flow chart] before, but I…don't use it. And I think it's largely because I suppose dealing with sore throats is something which perhaps I've felt as though I've gotten past the need to grab a decision support tool for, and it takes me more time to do.’ (GP1)*  *‘I'm wondering how much doctors have that perception, that that's how the patients will feel…And I've certainly tried encouraging some of the younger doctors…and medical students - it's really difficult to guess what this patient's attitude is. And I remember the first time, I was surprised when I thought I'm going to bite the bullet and tell them they don’t need antibiotics, and they said, “Oh, good, I was hoping you'd say that.”’ (GP1)*  Patient education and acceptance: managing patient expectations, myth busting  *‘I’m also twisting the narrative…“I need antibiotics to get better.” And I'll say, “Well, not necessarily, because you were born with this immune system, and that's actually what it's designed to do. And your immune system can make you better in the length of time that that antibiotic can do,”…you have to have a receptive audience.’ (PC1)*  *‘[Y]ou've got one group of patients who are really open to hearing those messages, and the others…that are absolutely dedicated to antibiotics as being the cornerstone of all treatment…they are definitely that much harder group to influence.’ (GP2)* |
| Therapeutic alliance: validation, personalised planning and symptomatic management |
| Building trust and relationships  ‘*I’m the person that…take(s) a collaborative approach…providing that menu of options…“hey, this is what I think would be suitable…Is that something that's going to work for you?”’ (GP5)*  *‘[To] build up a rapport…a trust with your doctor…in what he decides for you, because that's why you have chosen him. And if a 10-minute conversation with your doctor builds up a trust to say, “look, I don't want to take antibiotics unnecessarily”, then that's the way I feel about them.’(PT10)*  Telehealth: impact on clinical decision-making  ‘*My most recent [ARI] was a phone consult. And that adds that challenge…how do you do an adequate assessment over the phone? You can't check someone's oxygen saturations, you can't listen to their chest, you can't take a temperature, and you are relying on their capacity to provide that history to you.’ (GP5)*  Validation and reassurance  *‘Reassurance. That's exactly what people around our time in life want - that we are doing everything we can. And the doctor is doing everything…for our benefit and his benefit.’ (PT10)*  Individualised and personalised symptomatic management  *‘I wonder if…there (is) a patient handout that helps support the patient feeling held and nurtured but also helps the doctor…“here are the resources that you can use and here are the red flags that I'm concerned about….If these evolve for you, here's what you need to do”.’(GP5)*  *‘I've sometimes offered them a quick…Telehealth after-hours...I'll call them in two days…and just check on how they're going. If they say, “I'm definitely getting worse, I've got high fevers coughing out blood”, I'm going to say….“I'm sending a script…we'll do the examination in the next day or so”. That gives me a little bit of control there.’ (GP1).* |

*Table S 1. Theme 1: ‘Patient acceptance and engagement’ – a determinant of implementation of AMS interventions in general practice (continued)*

| Utility and accessibility of shared decision-making resources |
| --- |
| Decision-making in acute ARI  ‘*I could see…my patients just looking at the red dots [of SDM tool] and being, “[W]ell, obviously I'm sick enough to need antibiotics. I'm in that category; so gimmee, gimmee, gimmee”.’(GP4)*  *‘And if you're sick, your eyes probably couldn't focus on those jolly red and the blue dots [of SDM tool].’ (PT4)*  *‘[For the decision-aid to be useful]…first of all, the clinician would have to really recognise where their stance is early. So, if you were sitting at the 75 to 90% sure that they don't need antibiotics, then you might not engage with this process and just tell them like, “look, no, suck it up”. But if you were really on the 50% and you were comfortable coming to the conclusion of prescribing, then this process could work. It's tricky.’ (GP4)*  *‘[S]hared decision-making is ‘a bit of a buzz at the moment…most patients don't want to share decision-making. They come to the doctor, “I paid you doctor, tell me what to do”.’(GP1).*  Health literacy  *‘You’d need a significant degree of health literacy to be able to interpret this in an accurate way, which your average punter may not be up to the standard of being able to utilise it.’ (GP5)*  *‘Now, I can read graphs, and I understood what was going on, but a couple times you have to jump back…“which heading am I on again?” “Oh, this is the antibiotic, and the other one isn't the antibiotic”… I worked it out, but I don't know how many people would have the patience to see those three little circles…where there were complications caused by antibiotics.’ (PT2)* |
| Harm minimisation |
| Minimising economic and medical harm  *‘…I work with a lot of vulnerable populations that might not have a lot of cash…So, I put that out there as well, “…[an antibiotic] also costs you money. So, this is a way to use some things that you might have in the cupboard.”…It's about providing simple accessible options; they might not be able to make it to the chemist, and they might not get paid for a couple of days.’ (GP5)*  *‘I am aware of the cost issue and will often just [have] a quick bulk-billed [follow-up] Telehealth consultation…“I'm going to have a follow-up…I believe you, but I've got the back-up plan.”’ (GP01)*  *‘If I saw that [information about harms], I would take it very seriously on looking at the harms and I would ask about it and weigh it up.’ (PT8)*  *‘[W]e can counter that discussion a little bit by saying, “It might kill a bug, which you might not actually have, but it might make you a bit more susceptible to the other bugs”.’(GP2)*  *‘I would've preferred if she'd said, ‘I don't think you need antibiotics.’ I would've been happy to sort of go along with that…[my] concern was overuse. I don't like using antibiotics if it's not necessary. Because I am very well aware of the amount of overuse and the amount of resistance that's happening. Particularly in the hospitals, it's actually quite frightening. Because you'd expect, especially, to go to a hospital and be treated. And if you pick up something that's resistant to an antibiotic, well, you're in a bit of trouble.’(PT5)*  Delayed dispensing  *‘[“Delayed prescribing”] is when I feel I can't avoid giving them a script. This is the ‘harm minimisation’ route rather than the gold standard.’ (GP1)* |

ARI= Acute respiratory tract infection GP=General Practitioner PT = patient MB= microbiologist PC= Pharmacist PoCT = Point-of-care-testing CRP= c-reactive protein
