# Supplementary material for: Determinants of Implementation of Antimicrobial Stewardship Interventions for Managing Community Adult Acute Respiratory Infections: Qualitative Analysis from the OPTIMAS-GP Study Co-Design Phase
Source: Antibiotics (Basel). 2025 Sep 11;14(9):914. doi: 10.3390/antibiotics14090914 (PMC12466759; doi:10.3390/antibiotics14090914)
Supplement: Supplementary file 1 [file antibiotics-14-00914-s001.zip › Supplementary Table S2.docx]

*Table S2. Theme 2: ‘Practising within a system’ - a determinant of implementation of AMS interventions in general practice*

| Theme 2: PRACTISING WITHIN A SYSTEM |
| --- |
| Individual practitioners |
| Clinical acumen  *‘[My wife]’s father was on antibiotics. He only had to blow wind, and the doctor gave him antibiotics.’ (PT9)*  *‘I have noticed over time that antibiotics are sort of the ‘M&Ms’. They're just Band-Aids that they hand out I'm thinking maybe it would help…if they were put behind the glass door with the opiates and with a lock on them and said, “look, this is where they sit in our opinion”. You know, like, demonise them if you have to. They seem to be lollies.’(PT2)*  Utility of AMS resources  *’I’m using guidelines, typically the Therapeutic Guidelines, to try and grade the severity of illness, and signs and symptoms that might be pointing towards a viral or bacterial focus’. (MB)*  *‘I will actually routinely bring up [Therapeutic Guidelines] and show the patients what the rationale is, and I think patients find that useful…’ (GP4)*  Diagnostic uncertainty  *‘[I was] having a discussion with some of the other doctors today, noting how many cases of pneumonia that they've had recently…[I]n the last week and a half, we've both lost a patient who has died because of pneumonia…so that's probably a factor that has played on my mind a little bit in advising people “no, you don't need any antibiotics”…I think that can sometimes influence our prescribing patterns, just recent exposure to antibiotic-preventable illnesses which got antibiotics late and ended up in patients dying.’ (GP1)*  *‘Still, I'm quite fascinated by [PoCT-CRP]. I think that its utility more is in providing reassurance when it's negative.’ (MB)* |
| System influences |
| Continuity of care  *‘[Scheduling phone follow-up] was always possible to do, but it just is a lot easier now. The practice systems are set up around it more and patients are more familiar with that way of operating…’ [I say] “It would be pretty good to have a check in, and we can decide at that point whether the pattern is that I've anticipated for you or whether it's doing something different”.’ (GP6).*  *‘That's where [this town] has its own specific challenges because you actually only have the option of the urgent care clinics to be able to get seen within a two-week timeframe…no GPs are doing book-on-day appointments or urgent appointments at this stage.’ (GP5)*  Variations in practice  *‘[In a community pharmacy]…a patient came in with three different prescriptions, three different doctors, three different antibiotics, asking which one is the best for the cold she had.’ (PC2)*  *‘We certainly have a feeling it's something we talk about in the practice that Fridays are different from other days, and there is that element of the follow up arrangements are different… there are more acute respiratory presentations on the Friday.’ (GP6)* |

*Table S2. Theme 2: ‘Practising within a system’ - a determinant of implementation of AMS interventions in general practice (continued)*

| Adoption of AMS practices  *‘[T]he advantage of showing patients guidelines is that we're giving them a peek behind the curtain and being like, “These are the guidelines that we use every day. It's sacred”.’ (GP4)*  *‘I do see, when reviewing medications with patients [in general practice]…when I'm cleaning up a list, I will find Augmentin Duo Forte or something…on their list from years ago, and I'll say to the patient's doctor, “Take this off, because clearly that course is finished.” But sometimes it's a response from the doctor saying, “Oh, no, leave that there because that reminds me what to give them when they ask for something.” And, from the patient's point of view, they go, “Oh, no, no. I always have a script for that in the pocket, just in case”.’ (PC2*  *‘And, particularly with e-scripts, the doctors can literally cancel a script like that. But we're seeing them come up on the active script list from months ago…[it] would be good to encourage all GPs, if they've got access to those active script lists, to discontinue them, turn them off.’ (PC1)*  *‘We cane our registrars if they leave an antibiotic that's meant to have been a short term…on their patient’s current chart - So they learn very quickly, and all the software sets it up so it should just disappear. It's a once-only prescription. [T]here's really no excuse in this day and age.’ (GP2)*  Integration of PoCT of CRP into practice workflow  ‘*I'd probably want the patients doing that [prior rapid COVID and influenza testing before PoCT-CRP] outside of the practice. I just don't have the physical space in my clinic… And if they have to sit down for 20 minutes…I'd be thinking of from a practice flow point of view as well. Like, what happens with that time? Where does that sit within the consult or within the patient flow?’ (GP5)*  *‘The [practice nurse] could [do PoCT CRP], but if I've got a patient in my room and I decide it needs to be done, then I have to…ring the nurse say, ‘can you fit them in? If they can't - tell them to wait. The person who needs it is…unwell looking, and I guess it's then a matter of the nurse squeezing them in, bringing them into their room…Then me having to come across to the nurse's room and explain the results while I may have already started with another patient - maybe a knee deep in their depression, which is getting worse…That then holds the nurse up for another 15 minutes…So, there's all those other hidden costs other than just the process and it's very difficult in general practice to get it efficient in that way…Would I be prepared to take a hit of maybe $20 to convince the patient they don't need antibiotics? Probably not…But the other part of the cost is just the cost of having someone do it.’ (GP1)*  *‘I don't have a practice nurse so I would do it...as long as there wasn't some bulky machine that had to sit out for everyone to access, as long as it was like doing a pregnancy test or something like that, I can see myself doing it as part of the assessment and talking to the patient… while you're waiting for that two minutes to time in… So, I'm not sure that I would agree that it's too much to fit into one consult. I think if you knew that you had this thing available, and it did take two minutes, I think that it would be possible to weave it into that examination and bedside testing stage.’ (GP5)* |
| --- |

ARI= Acute respiratory tract infection GP=General Practitioner PT = patient MB= microbiologist PC= Pharmacist PoCT = Point-of-care-testing CRP= c-reactive protein
