# Supplementary material for: Determinants of Implementation of Antimicrobial Stewardship Interventions for Managing Community Adult Acute Respiratory Infections: Qualitative Analysis from the OPTIMAS-GP Study Co-Design Phase
Source: Antibiotics (Basel). 2025 Sep 11;14(9):914. doi: 10.3390/antibiotics14090914 (PMC12466759; doi:10.3390/antibiotics14090914)
Supplement: Supplementary file 1 [file antibiotics-14-00914-s001.zip › Supplementary Table S3.docx]

*Table S 3. Theme 3: ‘Prescribing stewardship’ - a determinant of implementation of AMS interventions in general practice*

| Theme 3: ‘PRESCRIBING STEWARDSHIP’ |
| --- |
| ‘Delayed prescribing’ as a concept |
| *‘I think mentally knowing I have it [an antibiotic prescription in case] in my back pocket, so if I do get worse, I don't have to go through the whole process again and I feel like I have a bit more control over it rather than not knowing when it will end.’ (PT7)*  *‘There's an honesty thing between you and the doctor that you're not getting the antibiotics for somebody else…then they don't have to go to the doctor's…So, you are playing doctor. So, there's a few little things that could come into it with deviant minds. Maybe I've got one of them.’ (PT10*) |
| ‘Delayed prescribing’ as an AMS intervention |
| ‘*I haven't had a huge amount [of patients] where they've gone, “I know you said don't fill this straight away, but I did”. I seem to have enough rapport to be able to say, look “yes, it will be tempting to fill this straight away, but we need to give your body a little bit more time”.’ (GP5)*  *‘There [are] people who say, “Oh, I got the script, but it actually wasn’t getting worse, so I didn't take them, but I kept the tablets just in case next time something happens.” And so, they're not even turning up at the pharmacy, saying, “I need this script, it’s six months old.” They've got it in their cupboard and then they're coming to see me saying, “Oh, I just had this infection. I took some antibiotics, got a little bit better. I suppose I just needed some more.” And, “who prescribed the antibiotics?” “You did. Six months ago”.’ (GP1)*  *‘I want to say “this has got to be not available to them anymore after 10 days…”…’Cause I think that is a system where we are really assured that those scripts don't just end up in circulation ongoing…it would help us do ‘delayed prescribing’ in a better way…if you give a script, it's not going to sit in the system forever.’ (GP2)* |
| Interprofessional collaboration |
| *‘[Dispensing instructions] at least to give…the poor old pharmacist some guidelines, because I can't imagine it's much fun when [the patients] say, “I'm not going to wait for this script. I might as well get it today”.’ (GP2)*  *‘I think a template on the script or a shortcut that you can store in your system. You just tick that particular comment [with expiry date] - is definitely the least ambiguous. It's understood by everyone, the patient who sees it written…pharmacist understands exactly what it means…’ (GP1)* |

ARI= Acute respiratory tract infection GP=General Practitioner PT = patient MB= microbiologist PC= Pharmacist PoCT = Point-of-care-testing CRP= c-reactive protein
