# Supplementary material for: Determinants of Implementation of Antimicrobial Stewardship Interventions for Managing Community Adult Acute Respiratory Infections: Qualitative Analysis from the OPTIMAS-GP Study Co-Design Phase
Source: Antibiotics (Basel). 2025 Sep 11;14(9):914. doi: 10.3390/antibiotics14090914 (PMC12466759; doi:10.3390/antibiotics14090914)
Supplement: Supplementary file 1 [file antibiotics-14-00914-s001.zip › Supplementary Table S4.docx]

*Table S4. Theme 4: ‘Diagnostic stewardship’ - a determinant of implementation of AMS interventions in general practice*

| Theme 4: ‘DIAGNOSTIC STEWARDSHIP’ |
| --- |
| Clinical acumen and decision-making |
| *‘[PoCT-CRP is] taking away from that clinical acumen that we're encouraging should be making the decisions rather than a test or anything else.’ (MB)*  *‘[C]linically, there's so many things to look out for respiratory that predict outcomes, but most of them are quite short term. There are…algorithms and stuff that you can use, say, in a patient with pneumonia to predict risk of complications. But, in terms of most of that, we do rely heavily on clinical decision. But I guess if you're worried about someone and you think you're going to start antibiotics, most of us would send them for bloods and an X-ray…if you're already sending them off for further tests, you're worried enough to do that, then give them antibiotics. I guess the question is, could CRP be helpful, if you're already going to send them off? (GP3)*  *‘‘By the time you get to doing that CRP, what proportion of your respiratory patients will you have already made that decision on? I'd be interested to see what proportion of people need a CRP and if you had a pretest decision about what you're going to do, how often would the CRP back it up? And how often does the CRP actually change what you do?’ (GP1)* |
| Overreliance on testing |
| *‘[I]t almost produces more investigation when we start with a diagnostic test like this. And I appreciate the other aspect is that we go and try and find the culprit. If we're saying it's a CRP, it's that middling ground, that might raise more concern about then finding an answer doing a full respiratory multiplex, which is very expensive and certainly isn't needed. So, it is then as was…brought up by [the GPs] when it was first suggested. Which was obviously it's taking away from that clinical acumen that we're encouraging should be making the decisions rather than a test or anything else.’ (MB)* |
| Result interpretation |
| *[C]onversely, if I had a completely clear chest X-ray, and a normal white cell count, is the CRP being elevated going to make me use antibiotics in that situation? Well, probably not.’ (GP5)*  *‘Yeah, and I think, like, I have seen cases where the CRP is much higher than you would expect it to be….But, I mean, for us, we would argue that it's a clinical decision terms of vitals and our assessment and all those sort of things.’ (GP3)* |
| Patient engagement and acceptance |
| *‘So, if it comes back as less than whatever it is on the algorithm, and then I say, “No antibiotics for you,” then that's helpful. If it comes back high, then we've just screwed ourselves, because, if I've got a really well-looking patient, and I'm happy with them, and I'm happy not prescribing, and then I do this test, then it railroads me into prescribing. So that's the gamble and, yeah, I don't think I'd be down for that.’ (GP4)*  *‘[I]'m very interested to see how it'll go [a study with PocT-CRP] because it's quite different…and the best aspect of it is the objective piece of information that the patient then can feel more trust in because they're lacking trust at the moment and they like a result. (MB)* |

ARI= Acute respiratory tract infection GP=General Practitioner PT = patient MB= microbiologist PC= Pharmacist PoCT = Point-of-care-testing CRP= c-reactive protein
