# Supplementary material for: Determinants of Implementation of Antimicrobial Stewardship Interventions for Managing Community Adult Acute Respiratory Infections: Qualitative Analysis from the OPTIMAS-GP Study Co-Design Phase
Source: Antibiotics (Basel). 2025 Sep 11;14(9):914. doi: 10.3390/antibiotics14090914 (PMC12466759; doi:10.3390/antibiotics14090914)
Supplement: Supplementary file 1 [file antibiotics-14-00914-s001.zip › Supplementary Table S5.docx]

*Table S5: Determinants of implementation of AMS interventions: themes and subthemes mapped to Theoretical Domains Framework (TDF) and COM-B components (Capability, Opportunity and Motivation)*

| CAPABILITY component (of COM-B) | | |  |
| --- | --- | --- | --- |
| Themes | Subthemes | Determinants of implementation of AMS interventions | TDF domains |
| Patient acceptance and engagement | Patient expectations | Pressures on GPs | Knowledge  Skills  Memory, attention and decision processes  Behavioural regulation |
|  |  | Managing expectations |  |
|  |  | Patient acceptance of education |  |
|  |  | Shared decision-making |  |
|  |  | Myth-busting |  |
|  | Therapeutic alliance: validation, personalised planning and symptomatic management | HCP and patient relationships |  |
|  |  | Building trust |  |
|  |  | Scheduling follow-up |  |
|  |  | Shared decision-making |  |
|  |  | Efficient personalised approach |  |
|  |  | Validation and reassurance |  |
|  |  | Symptomatic management |  |
|  |  | Scheduled follow-up reassessment |  |
|  |  | Time constraints |  |
|  | Utility and accessibility of shared decision-making resources | Utility in decision-making personalised planning, symptomatic management |  |
|  |  | Time commitment |  |
|  | Harm minimisation | Minimising economic and medical harm |  |
|  |  | Scheduling follow-up and Telehealth |  |
|  |  | Delayed prescribing |  |
| ‘Practising within a system’ | Individual practitioners | Clinical acumen |  |
|  |  | Outdated practices |  |
|  |  | Limited knowledge of resources |  |
|  |  | Diagnostic uncertainty |  |
|  |  | Individual response to PoCT of CRP use in general practice |  |
|  | System influences on patient-prescriber interaction | Continuity of care |  |
|  |  | Variations in ARI management |  |
|  |  | Scheduled follow-up appointments |  |
|  |  | Telehealth for follow-up |  |
|  |  | Peer-to-peer support |  |
|  |  | Audit and feedback |  |
|  |  | Training and supervision |  |
| ‘Prescribing stewardship’ | Delayed prescribing as a concept | Inconsistent messages |  |
|  |  | Patient reassurance |  |
|  | Delayed prescribing as a tool/AMS intervention | Patient uncertainty as to trigger |  |
|  |  | Adjunct patient advice |  |
|  | Interprofessional collaboration | Optimising ‘delayed prescribing’ strategy |  |
| ‘Diagnostic stewardship’ | Clinical acumen and decision-making | Confidence in clinical skills |  |
|  |  | Selective use of diagnostics |  |
|  | Overreliance on testing | Overreliance on diagnostic tests |  |
|  |  | Hindrance to skills acquisition |  |
|  | Result interpretation | Concern re missing diagnosis |  |
|  | Patient engagement and acceptance | Patient engagement |  |
| OPPORTUNITY component (of COM-B) | | | |
| Themes | Subthemes | Determinants of implementation of AMS interventions | TDF domains |
| Patient acceptance and engagement | Patient expectations | Managing expectations | Environmental context and resources  Social influences |
|  |  | Patient acceptance of education |  |
|  |  | Shared decision-making |  |
|  |  | Myth-busting |  |
|  | Therapeutic alliance: validation, personalised planning and symptomatic management | HCP and patient relationships |  |
|  |  | Building trust |  |
|  |  | Scheduling follow-up |  |
|  |  | Shared decision-making |  |
|  |  | Efficient personalised approach |  |
|  |  | Validation and reassurance |  |
|  |  | Symptomatic management |  |
|  |  | Scheduled follow-up reassessment |  |
|  |  | Telehealth and inability to physically examine |  |
|  |  | Time constraints |  |
|  | Utility and accessibility of shared decision-making resources | Utility for shared decision-making, personalised planning, symptomatic management |  |
|  |  | Time commitment |  |
|  |  | Health literacy |  |
|  | Harm minimisation | Loss of altruism |  |
|  |  | Minimising economic and medical harm |  |
|  |  | Scheduling follow-up and Telehealth |  |
|  |  | Delayed prescribing |  |
| ‘Practising within a system’ | Individual practitioners | Limited knowledge of resources |  |
|  | System influences on patient-prescriber interaction | Continuity of care |  |
|  |  | Scarce ‘book-on-day’ appointments |  |
|  |  | Second opinions and Urgent Care Centres |  |
|  |  | Scheduled follow-up appointments |  |
|  |  | Telehealth for follow-up |  |
|  |  | Peer-to-peer support |  |
|  |  | Audit and feedback |  |
|  |  | Training and supervision |  |
|  |  | Availability of AMS resources |  |
|  |  | Practice-wide approach to prescribing |  |
|  |  | PoCT of CRP integration into practice workflow |  |
| ‘Prescribing stewardship’ | Delayed prescribing as a concept | Inconsistent messages |  |
|  |  | GP shortages commensurate to patients’ needs |  |
|  |  | Patient reassurance |  |
|  | Delayed prescribing as a tool/AMS intervention | Antibiotic dispensing not as intended |  |
|  |  | Patient uncertainty as to trigger |  |
|  |  | Adjunct patient advice |  |
|  | Interprofessional collaboration | Practice (systems) approach to manage ‘delayed prescriptions’ |  |
|  |  | Optimising ‘delayed prescribing’ strategy |  |
| ‘Diagnostic stewardship’ | Overreliance on testing | Overreliance on diagnostic tests |  |
|  |  | Hindrance to skills acquisition |  |
|  | Patient engagement and acceptance | Patient engagement |  |
| MOTIVATION (component of COM-B) | | | |
| Themes | Subthemes | Determinants of implementation of AMS interventions | TDF domains |
| Patient acceptance and engagement | Patient expectations | Pressure on GPs | Beliefs about consequences  Professional role and identity |
|  |  | Managing expectations |  |
|  |  | Shared decision-making |  |
|  | Therapeutic alliance: validation, personalised planning and symptomatic management | HCP and patient relationships |  |
|  |  | Building trust |  |
|  |  | Scheduling follow-up |  |
|  |  | Shared decision-making |  |
|  |  | Efficient personalised approach |  |
|  |  | Validation and reassurance |  |
|  |  | Symptomatic management |  |
|  |  | Scheduled follow-up reassessment |  |
|  | Utility and accessibility of shared decision-making resources | Utility in decision-making, personalised planning, symptomatic management |  |
|  |  | Time commitment |  |
|  |  | Health literacy |  |
|  | Harm minimisation | Loss of altruism |  |
|  |  | Minimising economic and medical harm |  |
|  |  | Scheduling follow-up and Telehealth |  |
|  |  | Delayed prescribing |  |
| ‘Practising within a system’ | Individual practitioners | Clinical acumen |  |
|  |  | Outdated practices |  |
|  |  | Limited knowledge of resources |  |
|  |  | Diagnostic uncertainty |  |
|  |  | Individual response to PoCT of CRP use in general practice |  |
|  | System influences on patient-prescriber interaction | Scheduled follow-up appointments |  |
|  |  | Telehealth for follow-up |  |
|  |  | Peer-to-peer support |  |
|  |  | Audit and feedback |  |
|  |  | Training and supervision |  |
|  |  | PoCT of CRP integration into practice workflow |  |
| ‘Prescribing stewardship’ | Delayed prescribing as a concept | Inconsistent messages |  |
|  |  | Patient reassurance |  |
|  | Delayed prescribing as a tool/AMS intervention | Antibiotic dispensing not as intended |  |
|  |  | Adjunct patient advice |  |
|  | Interprofessional collaboration | Practice (systems) approach to manage ‘delayed prescriptions’ |  |
|  |  | Optimising ‘delayed prescribing’ strategy |  |
| ‘Diagnostic stewardship’ | Clinical acumen and decision-making | Confidence in clinical skills |  |
|  |  | Selective use of diagnostics |  |
|  | Overreliance on testing | Overreliance on diagnostic tests |  |
|  |  | Hindrance to skills acquisition |  |
|  | Result interpretation | Concern re missing diagnosis |  |

COM-B = Capability, Opportunity and Motivation – Behaviour change TDF = Theoretical Domains Framework AMS= Antimicrobial stewardship

GP=General Practitioner ARI= Acute respiratory tract infection PoCT = Point-of-care-testing CRP= C-reactive protein
